# Supplementary material for: Transcriptomic and Metabolomic Studies Reveal That Toll-like Receptor 2 Has a Role in Glucose-Related Metabolism in Unchallenged Zebrafish Larvae (Danio rerio)
Source: Biology (Basel). 2023 Feb 16;12(2):323. doi: 10.3390/biology12020323 (PMC9952914; doi:10.3390/biology12020323)
Supplement: Supplementary file 1 [file biology-12-00323-s001.zip › biology-2163195-supplementary.pdf]

Table S1. Sequences of primers used in qPCR reactions

Primers	Sequences	Application
gpib-F	5'...ATCATCGCATCCAAGACA...3'	qPCR
gpib-R	5'...GTGGCAGACCATAGGGAG...3'	qPCR
pfkma-F	5'...ATCCTGAACGTGGGTGCT...3'	qPCR
pfkma-R	5'...GTTCCCTGTCCAATCTC...3'	qPCR
pck2-F	5'...AGTGCTTTGCTCTTCGTATTG...3'	q-PCR
pck2-R	5'...CACCCTGAGGATTTGTGATTC...3'	qPCR
ppial-F	5'...TGAGCCGCAACAGTAATC...3'	Reference
ppial-R	5'...AAGGGAAAAGGAAGTGAAAG...3'	Reference

Table S2. 149 differential expressed genes (DEGs) in *tlr2*$^{-/-}$ versus *tlr2*$^{+/+}$ zebrafish larvae groups.

Ensembl ID	Gene name	log2 Fold Change	Svalue	zfin ID
ENSDARG00000111312	pabpc4	-11.50886308	4.85E-05	ZDB-GENE-030131-9663
ENSDARG00000094732	mical3b	-3.650047838	4.02E-13	ZDB-GENE-050211-1
ENSDARG00000086107	MTERF1	-2.608449278	6.87E-05	
ENSDARG00000097635	wu:fb18f06	-2.382020806	0.000325116	ZDB-GENE-030131-261
ENSDARG00000112325	ankrd29	-2.37796162	0.002841557	ZDB-GENE-050208-655
ENSDARG00000102185	PCDH8	-2.232158033	0.000494177	ZDB-GENE-120215-215
ENSDARG00000023900	casd1	-2.15233006	0.00012233	ZDB-GENE-060503-329
ENSDARG00000069085	ints2	-1.966907862	0.001856366	ZDB-GENE-050522-148
ENSDARG00000035043	pfdn5	-1.895289969	0.001392597	ZDB-GENE-030131-6858
ENSDARG00000062363	phex	-1.862050992	0.000367465	ZDB-GENE-030103-2
ENSDARG00000036076	heatr3	-1.847085627	0.004946941	ZDB-GENE-040426-1876
ENSDARG00000020956	pck2	-1.798342381	0.002386872	ZDB-GENE-040426-2266
ENSDARG00000007344	tcap	-1.724504939	0.001953038	ZDB-GENE-070501-5
ENSDARG00000069269	wdr35	-1.717885571	0.004152186	ZDB-GENE-060810-148
ENSDARG00000012140	ccnl1b	-1.697296922	9.27E-05	ZDB-GENE-030131-4813
ENSDARG00000020212	slc1a1	-1.63319199	0.001648019	ZDB-GENE-040718-414
ENSDARG00000074680	rims1a	-1.618739134	0.002337767	ZDB-GENE-090312-135
ENSDARG00000103826	gpib	-1.571970434	3.24E-05	ZDB-GENE-020513-3
ENSDARG00000093658	tm7sf3	-1.567456266	0.000265324	ZDB-GENE-030131-1770
ENSDARG00000041895	cad	-1.533319317	0.000229391	ZDB-GENE-021030-4
ENSDARG00000053222	asb5b	-1.518376915	0.00394881	ZDB-GENE-050417-271
ENSDARG00000042892	paip1	-1.51094519	0.000514962	ZDB-GENE-040801-247
ENSDARG00000027649	cpsf3	-1.453183989	0.002684177	ZDB-GENE-030131-3275
ENSDARG00000104835	map3k4	-1.434843344	0.000131865	ZDB-GENE-990603-4
ENSDARG00000039269	arg2	-1.434623812	0.000103138	ZDB-GENE-030131-1334
ENSDARG00000030537	psmc1a	-1.412975187	0.001100234	ZDB-GENE-030131-8730
ENSDARG00000040985	itgbl1	-1.408737717	0.00375369	ZDB-GENE-050522-410
ENSDARG00000008363	mcl1b	-1.389134145	0.001357441	ZDB-GENE-030825-1
ENSDARG00000004930	lmo7a	-1.339174696	0.000180495	ZDB-GENE-030219-74
ENSDARG00000026634	ehmt1b	-1.32028779	0.001462314	ZDB-GENE-080515-3
ENSDARG00000093768	prr18	-1.307904661	0.000303383	ZDB-GENE-081107-6
ENSDARG00000116161	trip12	-1.300353357	0.002435134	ZDB-GENE-041111-262
ENSDARG00000002745	tdh	-1.297655367	1.15E-05	ZDB-GENE-040426-2379
ENSDARG00000090369	zgc:86896	-1.275307853	2.78E-05	ZDB-GENE-040625-80
ENSDARG00000020711	rrm2	-1.259997735	0.001812058	ZDB-GENE-990415-25
ENSDARG00000104028	si:dkey-74k8.3	-1.213805257	0.001323098	ZDB-GENE-141222-20
ENSDARG00000071445	myoz1b	-1.193692113	0.001193759	ZDB-GENE-040718-146
ENSDARG00000101362	mibp	-1.16819841	0.002001775	ZDB-GENE-030404-1
ENSDARG00000076066	march6	-1.05686572	0.000976824	ZDB-GENE-070912-530

ENSDARG00000014179	pfkma	-1.054113785	0.00362629	ZDB-GENE-040912-135
ENSDARG00000076667	ccng1	-1.029350676	0.000203098	ZDB-GENE-020322-1
ENSDARG00000059070	gars	-0.985407815	0.003399213	ZDB-GENE-030131-9174
ENSDARG00000007216	abce1	-0.980665904	0.002288344	ZDB-GENE-040426-1995
ENSDARG00000009136	tp53bp2a	-0.978283727	0.00456419	ZDB-GENE-040516-8
ENSDARG00000104267	postnb	-0.954685124	0.001728197	ZDB-GENE-030131-9120
ENSDARG00000037559	uba1	-0.951795928	0.002098331	ZDB-GENE-040426-2009
ENSDARG00000070043	dars	-0.949478772	0.000584858	ZDB-GENE-061110-135
ENSDARG00000005908	clk4b	-0.938504204	0.004636476	ZDB-GENE-050227-19
ENSDARG00000039502	eef1a1a	-0.92364574	0.003097816	ZDB-GENE-030131-8278
ENSDARG00000035869	azin1b	-0.917667197	0.003509696	ZDB-GENE-030121-1
ENSDARG00000102415	scinla	-0.888267996	0.002145779	ZDB-GENE-030131-2005
ENSDARG00000032575	ywhaz	-0.886185801	0.001426919	ZDB-GENE-030131-8554
ENSDARG00000014763	arf2a	-0.874375665	0.001039742	ZDB-GENE-040122-4
ENSDARG00000011510	rcc2	-0.864013114	0.002789533	ZDB-GENE-040426-2213
ENSDARG00000036427	slc3a2a	-0.862452704	0.001534392	ZDB-GENE-000831-3
ENSDARG00000012505	u2af2a	-0.859597877	0.003296305	ZDB-GENE-050706-131
ENSDARG00000013755	actn3a	-0.846336643	0.001008642	ZDB-GENE-000329-9
ENSDARG00000006766	snd1	-0.835038259	0.002240737	ZDB-GENE-030131-3124
ENSDARG00000053810	hnrnpc	-0.811196091	0.004792526	ZDB-GENE-040426-2043
ENSDARG00000009881	ier5	-0.795401148	0.003452196	ZDB-GENE-030616-127
ENSDARG00000044526	camk1ga	0.722898297	0.004287985	ZDB-GENE-030131-7594
ENSDARG00000103498	epd	0.835312342	0.004084159	ZDB-GENE-980526-111
ENSDARG00000016181	trim33	0.859464059	0.002893176	ZDB-GENE-030131-2773
ENSDARG00000078567	lonrf1l	0.891343304	0.004355802	ZDB-GENE-081104-397
ENSDARG00000056511	arr3a	0.907218606	0.00075928	ZDB-GENE-040718-102
ENSDARG00000099380	rpl13	0.913049041	0.004220261	ZDB-GENE-031007-1
ENSDARG00000008861	tfap2e	0.913995012	0.000822459	ZDB-GENE-040426-1455
ENSDARG00000015222	cbll1	0.93711543	0.002484589	ZDB-GENE-040426-691
ENSDARG00000022767	apobb.1	0.948168919	0.001688517	ZDB-GENE-030131-9732
ENSDARG00000044852	wbp2nl	0.951956769	0.003567714	ZDB-GENE-030131-4012
ENSDARG00000044684	rbp4l	0.956030354	0.003883762	ZDB-GENE-030131-7591
ENSDARG00000101637	ccnd1	0.958626191	0.00334795	ZDB-GENE-980526-176
ENSDARG00000105154	creg1	0.958820675	0.004714576	ZDB-GENE-070112-2042
ENSDARG00000077934	tegt	0.965657623	0.000853318	ZDB-GENE-030826-10
ENSDARG00000102147	fem1c	0.976692308	0.004015451	ZDB-GENE-031008-3
ENSDARG00000104316	CABZ01078055.1	0.981499354	0.003690451	
ENSDARG00000014717	dync1h1	1.01263213	0.002584147	ZDB-GENE-030131-7050
ENSDARG00000042458	rfc4	1.01482853	0.003197451	ZDB-GENE-040824-3
ENSDARG00000038028	ndufa6	1.023404737	0.003046918	ZDB-GENE-040426-1124
ENSDARG00000099640	eed	1.040600594	0.001570851	ZDB-GENE-050417-287
ENSDARG00000101180	mcm7	1.041069767	0.002736753	ZDB-GENE-020419-27
ENSDARG00000042535	actc1a	1.049163287	0.004495255	ZDB-GENE-040520-4
ENSDARG00000087206	cct2	1.057455245	0.003818091	ZDB-GENE-020419-6
ENSDARG00000039302	terfa	1.064344931	0.003246386	ZDB-GENE-020419-38
ENSDARG00000032296	pomp	1.064403344	0.001162289	ZDB-GENE-040801-10
ENSDARG00000099406	lrit1b	1.066999323	0.001608373	ZDB-GENE-060616-45
ENSDARG00000067966	fem1a	1.077436053	0.004425456	ZDB-GENE-030131-5824
ENSDARG00000089663	lsm3	1.082090359	0.003147929	ZDB-GENE-161207-2
ENSDARG00000087873	eevs	1.09985931	0.000656731	ZDB-GENE-131121-365
ENSDARG00000002193	rho	1.100194463	0.001767905	ZDB-GENE-990415-271
ENSDARG00000090389	ndufv3	1.121415365	0.002050292	ZDB-GENE-030131-6500
ENSDARG00000006345	med17	1.127559972	0.000731888	ZDB-GENE-040302-1
ENSDARG00000075201	inpp4b	1.128604718	0.000883321	ZDB-GENE-090312-97
ENSDARG00000076618	tm2d3	1.152316283	0.000408743	ZDB-GENE-070620-20

ENSDARG00000052840	ndufs4	1.16071928	7.62E-05	ZDB-GENE-050522-421
ENSDARG00000102558	pde6ha	1.165457384	0.00070644	ZDB-GENE-040426-1754
ENSDARG00000040557	exosc5	1.176202152	0.002634434	ZDB-GENE-060503-675
ENSDARG00000011125	snrpb	1.204146061	0.000681834	ZDB-GENE-040426-1819
ENSDARG00000036359	riox2	1.214369629	0.002193604	ZDB-GENE-040426-1283
ENSDARG00000039934	hlcs	1.224335366	0.001130432	ZDB-GENE-030131-5333
ENSDARG00000098934	rrp7a	1.226532022	0.000140871	ZDB-GENE-050417-38
ENSDARG00000103099	EARS2	1.231474585	0.000388234	ZDB-GENE-060825-214
ENSDARG00000092787	si:dkey-88p24.11	1.259627739	0.002944792	ZDB-GENE-030131-8991
ENSDARG00000086453	cx52.9	1.264161403	0.002995968	ZDB-GENE-040426-2421
ENSDARG00000070386	krtcap2	1.272348773	0.000791182	ZDB-GENE-060825-91
ENSDARG00000086411	srsf10b	1.282514336	0.000538685	ZDB-GENE-040426-1415
ENSDARG00000014556	serpinb1l3	1.287112273	0.000428599	ZDB-GENE-030131-7059
ENSDARG00000063914	mt-nd3	1.296136716	0.000215024	ZDB-GENE-011205-9
ENSDARG00000075883	bub3	1.302003039	6.11E-05	ZDB-GENE-041010-210
ENSDARG00000101347	CABZ01068251.1	1.308592166	0.000191176	
ENSDARG00000025581	rpl10	1.318690312	0.000346299	ZDB-GENE-030131-8656
ENSDARG00000069109	sssca1	1.330924829	0.002533847	ZDB-GENE-041010-102
ENSDARG00000100409	camlg	1.335267365	0.000472736	ZDB-GENE-040426-2407
ENSDARG00000089362	grn1	1.423718291	0.001224971	ZDB-GENE-060103-1
ENSDARG00000013726	ap4b1	1.435379757	0.000112923	ZDB-GENE-040426-1284
ENSDARG00000026759	ldlrb	1.46417277	3.71E-05	ZDB-GENE-040426-1254
ENSDARG00000029204	tyrp1a	1.474365614	2.00E-05	ZDB-GENE-070718-2
ENSDARG00000013921	frya	1.536832985	0.000913177	ZDB-GENE-060510-4
ENSDARG00000087401	slc25a34	1.589549351	0.000149647	ZDB-GENE-040426-1442
ENSDARG00000090544	CABZ01085658.1	1.627872459	0.000169904	
ENSDARG00000077368	slc30a6	1.660849318	1.44E-05	ZDB-GENE-040426-1838
ENSDARG00000103464	pggt1b	1.703944502	3.57E-07	ZDB-GENE-050913-85
ENSDARG00000020239	lpin1	1.717408873	0.001070018	ZDB-GENE-080722-2
ENSDARG00000046024	pym1	1.755277237	2.14E-07	ZDB-GENE-040426-1464
ENSDARG00000017367	rhbdf1b	1.836021455	1.07E-07	ZDB-GENE-130531-6
ENSDARG00000113649	actb1	1.881919676	1.71E-05	ZDB-GENE-000329-1
ENSDARG00000041304	trak1a	1.950389019	0.000944202	ZDB-GENE-100922-182
ENSDARG00000020845	tns1b	2.050211491	0.00015886	ZDB-GENE-030131-6933
ENSDARG00000070834	taf13	2.116550061	3.38E-09	ZDB-GENE-030131-2873
ENSDARG00000111458	wu:fi09b08	2.216227623	0.001256655	ZDB-GENE-030131-5630
ENSDARG00000105341	si:dkey-9l20.3	2.322966385	4.15E-05	ZDB-GENE-090313-369
ENSDARG00000101799	si:ch1073-82l19.1	2.335118093	0.004870059	ZDB-GENE-030131-5941
ENSDARG00000112284	AL935186.7	2.51913611	8.50E-06	
ENSDARG00000116076	FO704772.2	3.346671885	2.17E-06	
ENSDARG00000012306	syt13	3.673025499	0.000450768	ZDB-GENE-050417-135
ENSDARG00000038728	ch25hl2	4.274294692	0.001498134	ZDB-GENE-080204-82
ENSDARG00000057143	nradd	5.252442372	0.000608766	ZDB-GENE-030131-2537
ENSDARG00000076104	sema4bb	5.545730168	0.001289387	ZDB-GENE-120503-1
ENSDARG00000094605	si:ch211-154e10.1	5.657330342	8.43E-05	ZDB-GENE-081104-136
ENSDARG00000079884	trim107	6.043260797	0.000282885	ZDB-GENE-110919-5
ENSDARG00000117149	neurod4	8.602986421	7.78E-08	ZDB-GENE-030730-1
ENSDARG00000052610	olig4	8.842947263	3.61E-08	ZDB-GENE-030131-3580
ENSDARG00000109439	gucy1a1	8.879578498	0.001903355	ZDB-GENE-050417-230
ENSDARG00000115893	BX004787.1	9.71693913	0.000631992	
ENSDARG00000058464	rasd3	9.759533075	0.000562081	ZDB-GENE-060818-9
ENSDARG00000114428	slc35c2	10.28750789	0.000247448	ZDB-GENE-030131-2202
ENSDARG00000115748	BX322566.3	11.30635592	5.49E-05	
ENSDARG00000115484	clta	12.13298202	2.31E-05	ZDB-GENE-040426-1986
ENSDARG00000110705	srsf7a	12.9219612	4.89E-06	ZDB-GENE-040426-1798

Table S3. Top 10 significantly enriched pathways in *tlr2*$^{+/+}$ larvae by GSEA analysis

Name	Systematic name	NES	FDR q-value	Description
KEGG_VALINE_LEUCINE_AND_ISOLEUCINE_DEGRADATION	M11835	-3.18239	0	Amino acid metabolism
KEGG_GLYCOLYSIS_GLUCONEOGENESIS	M11521	-3.06106	0	Carbohydrate metabolism: Glycolysis is the process of converting glucose into pyruvate and generating small amounts of ATP (energy) and NADH (reducing power).
WP_TRANSLATION_FACTORS	M3942	-2.99138	7.41E-04	Protein synthesis is the ultimate step of gene expression and a key control point for regulation. In particular, it enables cells to rapidly manipulate protein production without new mRNA synthesis, processing, or export. This pathway gives an overview of the translation factors involved in this process.
SHEPARD_CRASH_AND_BURN_MUTANT_UP	M1082	-2.91601	0.003629	Human orthologs of BMYB target genes in zebra fish, identified as commonly changed in the BMYB loss of function mutant crb ('crush and burn') and after knockdown of BMYB by morpholino.
RHEIN_ALL_GLUCOCORTICOID_THERAPY_DN	M1859	-2.8598	0.007154	Genes down-regulated in ALL (acute lymphoblastic leukemia) blasts after 1 week of treatment with glucocorticoids.
MARKEY_RB1_ACUTE_LOF_UP	M15606	-2.78584	0.014707	Genes up-regulated in adult fibroblasts with inactivated RB1 by Cre-lox: acute loss of function (LOF) of RB1.
WP_TRYPTOPHAN_METABOLISM	M39500	-2.76486	0.015475	This pathway describes the metabolism of tryptophan, an essential amino acid.
KEGG_FATTY_ACID_METABOLISM	M699	-2.7604	0.013819	Lipid metabolism
MANALO_HYPOXIA_DN	M18562	-2.74929	0.013652	Genes down-regulated in response to both hypoxia and overexpression of an active form of HIF1A.
KEGG_PYRUVATE_METABOLISM	M7934	-2.72468	0.015652	Carbohydrate metabolism

Note: NES: Normalized Enrichment Score. FDR q-value: False Discovery Rate method of correction for multiple testing. Setting the FDR q-value < 0.01 as the cutoff to find significantly enriched gene sets.

Table S4. Top 10 significantly enriched pathways in *tlr2*$^{-/-}$ larvae by GSEA analysis

Name	Systematic name	NES	FDR q-value	Description
WP_CYTOPLASMIC_RIBOSOMAL_PROTEINS	M39495	5.488139	0	Translation
KEGG_RIBOSOME	M189	5.351484	0	Ribosome
REACTOME_SELENOAMINO_ACID_METABOLISM	M27170	5.340363	0	Metabolism Selenoamino acids
REACTOME_SRP_DEPENDENT_COTRANSLATIONAL_PROTEIN_ TARGETING_TO_MEMBRANE	M567	5.257831	0	Translation
REACTOME_EUKARYOTIC_TRANSLATION_ ELONGATION	M29556	5.246786	0	Translation
REACTOME_RESPONSE_OF_EIF2AK4_GCN2_TO_ AMINO_ACID_ DEFICIENCY	M27686	5.224345	0	Cellular response to starvation
REACTOME_INFLUENZA_INFECTION	M4669	5.158294	0	Infectious disease
REACTOME_NONSENSE_MEDIATED_DECAY_NMD	M1067	4.971412	0	The Nonsense-Mediated Decay (NMD) pathway activates the destruction of mRNAs containing premature termination codons (PTCs)
REACTOME_EUKARYOTIC_TRANSLATION_ INITIATION	M27686	4.818882	0	Translation
REACTOME_REGULATION_OF_EXPRESSION_OF _SLITS_AND_ ROBOS	M27876	4.58654	0	Expression of SLIT and ROBO proteins is regulated at the level of transcription, translation and protein localization and stability.

Note: NES: Normalized Enrichment Score. FDR q-value: False Discovery Rate method of correction for multiple testing. Setting the FDR q-value < 0.01 as the cutoff to find significantly enriched gene sets.

Table S5. 29 differentially expressed metabolites in *tlr2*$^{-/-}$ versus *tlr2*$^{+/+}$ zebrafish larvae groups.

HMDB ID	Metabolite name	log2 Fold Change	*P* value
HMDB0001389	Melatonin	-0.870716983	0.00737003
HMDB0000139	Glycerate	-0.730487799	0.0170277
HMDB0000123	Glycine	0.501108449	0.0212432
HMDB0000650	2-Aminobutyrate	0.647698256	0.0438577
HMDB0000684	Kynurenine	0.838719093	0.0325465
HMDB0000254	Succinate	0.849757364	0.0134592
HMDB0000362	2-Phosphoglycerate	0.906744196	0.00293618
HMDB0001487	NADH	1.053637964	0.00429618
HMDB0000122	Glucose	1.150958365	0.0162585
HMDB0000197	Indole-3-acetate	1.172639386	0.000367466
HMDB0000224	O-Phosphoethanolamine	1.227068909	0.00368356
HMDB0000112	4-Aminobutyrate	1.237098354	0.000957455
HMDB0000687	Leucine	1.285589984	0.00402587
HMDB0033780	Asparagine	1.36962882	0.00999398
HMDB0000042	Acetate	1.478717078	0.0205411
HMDB0000191	Aspartate	1.557556343	0.00267089
HMDB0304356	Formate	1.563109087	0.0116735
HMDB0000143	Galactose	1.590993483	0.0118101
HMDB0000148	Glutamate	1.723693237	0.00287398
HMDB0000167	Threonine	1.833003509	0.00334483
HMDB0000883	Valine	1.924776666	0.000342496
HMDB0000161	Alanine	2.054707706	0.000136682
HMDB0003423	Glutamine	2.186947544	0.000247798
HMDB0000158	Tyrosine	2.388317711	0.0184276
HMDB0000190	Lactate	2.446310033	0.00222552
HMDB0000149	Ethanolamine	2.629859039	0.00782938
HMDB0000064	Creatine	2.655119001	< 0.0001
HMDB0000156	Malate	4.375734539	0.0002337
HMDB0000251	Taurine	4.91040508	< 0.0001
